# Challenges on the provision of palliative care for patients with cancer in low- and middle-income countries: a systematic review of reviews

**DOI:** 10.1186/s12904-020-00558-5

**Published:** 2020-04-22

**Authors:** Hammoda Abu-Odah, Alex Molassiotis, Justina Liu

**Affiliations:** School of Nursing, The Hong Kong Polytechnic University, Kowloon, Hong Kong

**Keywords:** Cancer, Challenges to care, Low-income countries, Middle-income countries, Palliative care

## Abstract

**Background:**

Despite the significant benefits of palliative care (PC) services for cancer patients, multiple challenges hinder the provision of PC services for these patients. Low- and middle-income countries (LMICs) are witnessing a sharp growth in the burden of non-communicable diseases. There is a significant gap between demand and supply of PC in LMICs in current health services. This review aims to synthesise evidence from previous reviews and deliver a more comprehensive mapping of the existing literature about personal, system, policy, and organisational challenges and possible facilitators on the provision of PC services for cancer patients in LMICs.

**Methods:**

A systematic review of reviews was performed following PRISMA guidelines. PubMed, EMBASE, SCOPUS, PsycINFO, Web of Sciences, CINAHL, and Cochrane Library databases were searched to identify review papers published between 2000 and 2018 that considered challenges and possible facilitators to PC provision. A modified socioecological model was used as a framework for analysing and summarising findings.

**Results:**

Fourteen reviews were included. The reviews varied in terms of aim, settings, and detail of the challenges and possible facilitators. The main challenges of personal and health care systems included knowledge deficits and misunderstandings from patients, families, the general public, and health care providers about PC; and inadequate number of trained workforce. Besides, limited physical infrastructure, insufficient drugs for symptom relief and lack of a comprehensive national plan for implementing PC were the core organisational and policy level challenges that were recognised. Furthermore, the main possible facilitators that were identified included provision of adequate training for health care providers and health education for patients, families and the general public to enhance their knowledge, beliefs, and attitudes to PC. Finally, involvement of policymakers and making drugs available for symptom relief should also be in place to improve the health care systems.

**Conclusions:**

Understanding challenges to the provision of PC for people with cancer could help in the development of a PC pathway in LMICs. This knowledge could be used as a guide to develop an intervention programme to improve PC. Political influence and support are also required to ensure the sustainability and the provision of high-quality PC.

## Background

Cancer includes more than 100 different diseases of unknown aetiology [1]. It is an increasingly significant reason for morbidity and mortality all over the world [2]. In 2018, about 18.1 million new cases of cancer were diagnosed globally [3]. In the same year, cancer accounts for about 9.6 million deaths; 70% of deaths are registered in low- and middle-income countries [3]. Low-and middle-income countries (LMICs), as defined by the World Bank, are countries whose Gross National Income ranges between $996 and $3895 [4]. These countries are experiencing an increase in the burden of non-communicable diseases, including cancer [3]. By 2035, about two-thirds of new cancer cases will be diagnosed in LMICs [5]. This will put enormous pressures and strain on the health care systems of LMICs [6] as most of these countries are not well-prepared and organised to manage this growing burden and suffer from insufficient budget allocation and limited resources [7]. Treating such diseases place high demands on health services in countries with scarce resources resulting in high direct and indirect costs of care.

The consequences of cancer and its treatments have been significant on the quality of life (QOL) of patients and their families [8, 9]. The diagnosis of cancer frequently results in a complex set of issues that patients and their families must confront [10]. Alleviating the consequences of cancer and providing high-quality of care, including symptom management, handling side effects, as well as social, psychological, spiritual and emotional support are recognised as high priority aspects that should be taken into account [11]. These aspects are categorised under the PC umbrella [12, 13].

Palliative care (PC) is considered one of the most holistic and appropriate approaches to provide specialised medical and nursing care for patients with chronic illnesses [14] and makes the lives of patients with advanced diseases to be meaningful and productive. The 2002 WHO definition of PC states that “*PC is an approach that improves the quality of life of patients and their families through the prevention and relief of suffering by means of early identification and impeccable assessment and treatment of pain and other problems, physical, psychosocial and spiritual*” [12, 13, 15] (p.84, ref. [15]). PC is intended to relieve symptoms that appear when cancer is progressing and allow patients to live comfortably rather than cure the disease [3, 16]. Due to a transitional demographic change in population growth in the world and increase in life expectancy, the need for PC has increased, and the application of this approach is urgently required to be adopted [17].

Palliative care is a holistic approach focusing on all patients with all incurable diseases [18, 19], but PC programs in many countries start with cancer patients as this is the largest group of of patients with life-limiting diseases and are often admitted at the hospital for an extended period [20]. Long term admissions create pressures on a country’s health care system especially when confined with a lack of budget allocation, limited resources, and lack advanced technologies for cancer detection and treatment [7]. Almost all of the cancer patients in LMICs are diagnosed at a late stage, making them more inclined to experience severe pain and distress [21, 22], respiratory and gastrointestinal problems, and loss of consciousness, all related to disease progression [23]. Considering that, patients with cancer are in a high priority in need of PC at the end-stage-of-life. Also, being the largest group, it is often the focus when countries develop their PC programmes, before introducing it into other groups of patients with end of life and PC needs [24]. While it is acknowledged that there is an ethical imperative to provide PC for all patients with incurable and life-limiting illnesses and that the PC priorities, particularly in the African continent, may be related more to treating patients with HIV/AIDS and related comorbidities and co-diseases, the focus of this paper is on cancer patients for two reasons. Firstly, cancer rates, particularly in Africa, are expected to grow by 400% over the next 50 years [25], 70% of cancer deaths take place in LMICs and 70% of patients diagnosed with cancer in LMICs are diagnosed at a very advanced stage [2, 3, 26]. Secondly, this review is an initial stage of a larger project that is developed to address some of the key issues around implementing a PC programme in Palestine. In the context of Palestine, cancer is the second leading cause of death (at 14%) with an expected high increase in the cancer burden that will create challenges in the delivery of care to patients that are mostly diagnosed at a late stage [27]. The African Palliative Care Association has recently also advocated for more PC to be provided to cancer patients and more access to opioids and other essential medicines [28].

Although PC has become a significant approach to improving the QOL of patients worldwide, only 3 million out of 20 million patients with a life-threatening illness in the world receive PC services. Most of these services are available and provided in developed countries [24]. In many LMICs, PC services are not available, and this is attributed to multiple challenges that continue to create obstacles to their availability and implementation. For instance, most PC models that exist are developed and implemented in Western countries [29, 30], and may not be congruent with some cultural issues (e.g., religion, beliefs, and norms) in other countries. Health care provider (HCP)-related issues, such as training and education, are other obstacles preventing the adoption of PC [31–34] in LMICs. Besides, related administrative matters, such as access to opioids [31, 32, 35] and unwillingness of patients and families to be referred to specialised PC units may also prevent the adoption of PC services.

Some action measures have been undertaken by WHO to promote and implement PC in the health care systems of LMICs [36]. However, these measures have faced many obstacles during implementation. LMICs are experiencing a significant gap between demand and supply of PC services, and therefore, immediate actions are required to overcome these impediments [37]. The urgency is heightened by the fact that most cancer patients in LMICs are diagnosed during the late-stage of the disease and, therefore, they are in desperate need of adequate PC [38].

A number of studies have been conducted worldwide to assess the challenges of dispensing PC services. Donkor et al., [39] assessed challenges in LMICs, Fadhil et al., [40] focused on similar issues in the Eastern Mediterranean region, and Aldridge et al., [41] focused on challenges to integrating PC in the USA. These studies focused generally on patients with life-threatening diseases with limited attention given to cancer patients. However, no systematic overview synthesising the challenges and possible facilitators on the provision of PC in LMICs has been reported.

**Fig. 1:**
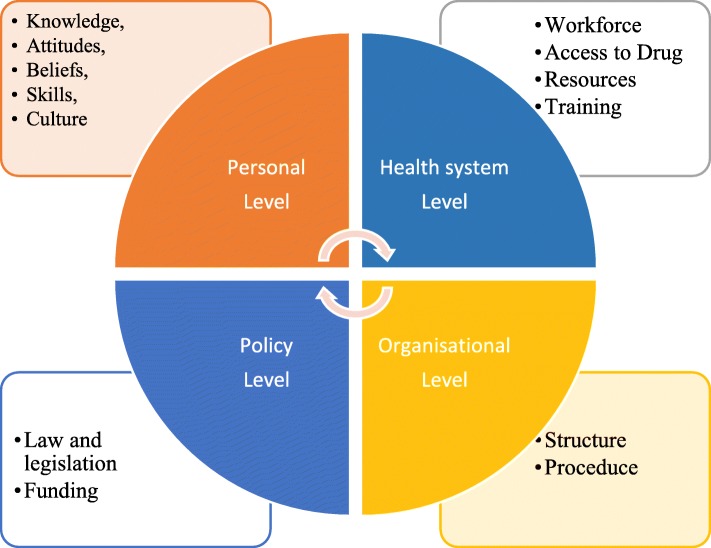
A modified socioecological framework [42]

Therefore, this current systematic review is conducted to synthesise evidence from previous literature and provide a comprehensive mapping of the existing literature about challenges and possible facilitators in the provision of PC for cancer patients in LMICs. The methodology utilised is based on the socio-ecological model (SEM) [42]. This model is widely used across studies for having multilevel determinants [43, 44]. It has four levels; personal level; organisational level; health system level; and policy/payment level (Fig. 1). An intensive and comprehensive search of seven databases has been carried out with focus on patients with cancer in LMICs. This paper seeks to answer the following questions: (1) what are the challenges associated with the provision of PC for cancer patients in LMICs? And (2) what are the possible facilitators that can overcome these challenges?

## Methods

### Study design

This is a systematic review of reviews aimed at providing a broad overview of the field, and mapping the current body of work on challenges of providing PC services to cancer patients. This review is structured in accordance with the PRISMA (preferred reporting items for systematic reviews and meta-analyses) guidance [45].

### Search sources and strategies

Searches were performed on seven electronic databases: PubMed, EMBASE, SCOPUS, PsycINFO, Web of Sciences, Cumulative Index to Nursing and Allied Health Literature (CINAHL), and Cochrane Database of Systematic Reviews. The search was specific to review articles published in English language in or after 2000 to capture information more relevant to current health care systems, and given that PC is defined by WHO in 1998 and optimised in 2002 [12].

The search strategy of this systematic review is based on the PCC (population, concept, and context) framework. The following terms are included in the search strategy; first, terms for “challenges”, such as “barriers”, “problems”, “limitations”, and “obstacles” were included. Second, words synonymous to “provision”, such as ‘bringing”, and “access” were also added in the search. Third, terms like “palliative care”, such as “palliative medicine”, “hospice care”, “supportive care”, “terminal care”, and “end-of-life care” were further added. Fourth, terms for “cancer”, such as “tumor”, “neoplasms”, “terminal cancer”, “metastatic cancer” and “malignant” were included. Finally, expressions for “review*” were included. All these terms were linked using the Boolean operator “AND” and Medical Subject Heading (MeSH) terms were used. Table 1 shows the detailed search terms for PubMed and CINAHL databases adjusted appropriately for the other databases.

**Table 1:** Selected Search Strategies for review articles

Search	Search Term	Hits
PubMed		
S1	Search ((((challenges [Title/Abstract]) OR obstacles [Title/Abstract]) OR limitations [Title/Abstract]) OR problems [Title/Abstract]) OR barriers [Title/Abstract]	1,051,391
S2	Search ((provision [Title/Abstract]) OR bringing [Title/Abstract]) OR access [Title/Abstract]	350,573
S3	Search (((((palliative care [MeSH Terms]) OR palliative medicine [Title/Abstract]) OR hospice care [Title/Abstract]) OR supportive care [Title/Abstract]) OR terminal care [Title/Abstract]) OR end of life care [Title/Abstract]	72,182
S4	Search (((((cancer [Title/Abstract]) OR tumor [Title/Abstract]) OR neoplasms [Title/Abstract]) OR terminal cancer [Title/Abstract]) OR metastatic cancer [Title/Abstract]) OR malignant [Title/Abstract]	2,462,108
S5	Search review*[Title/Abstract] Sort by: Best Match	1,878,924
S6	S1 AND S2 AND S3 AND S4 AND S5	88
S7	S1 AND S2 AND S3 AND S4 AND S5 (Limiters - Date of Publication: 20000101–20,190,110; English Language)	79
CINAHL		
S1	TI challenges OR TI barriers OR TI problems OR TI limitations OR TI obstacles	68,419
S2	AB challenges OR AB barriers OR AB problems OR AB limitations OR AB obstacles	373,747
S3	AB provision OR AB bringing OR AB access	110,926
S4	TI provision OR TI bringing OR TI access	30,549
S5	TI palliative care OR TI palliative medicine OR TI hospice care OR TI supportive care OR TI terminal care OR TI end of life care	22,990
S5	AB palliative care OR AB palliative medicine OR AB hospice care OR AB supportive care OR AB terminal care OR AB end of life care	25,576
S7	AB cancer OR AB tumor OR AB neoplasms OR AB terminal cancer OR AB metastatic cancer OR AB malignant	270,124
S8	TI cancer OR TI tumor OR TI neoplasms OR TI terminal cancer OR TI metastatic cancer OR TI malignant	252,261
S9	TI review*	176,934
S10	AB review*	366,746
S11	S1 OR S2	416,347
S12	S3 OR S4	130,606
S13	S5 OR S6	37,837
S14	S7 OR S8	392,676
S15	S9 OR S10	473,045
S16	S11 AND S12 AND S13 AND S14 AND S15	74
S17	S11 AND S12 AND S13 AND S14 AND S15 (Limiters - Date of Publication: 20000101–20,190,110; English Language)	71

Explanation of abbreviations: *S* Search; *MeSH* Medical Subject Headings; *AB* Abstract; *TI* Title

### Criteria for considering studies in this review

#### Inclusion criteria

- Review articles only;
- Review focusing on patients diagnosed with cancer;
- Review focusing on LMICs;
- Review focusing on patients aged at least 18 years, or the words ‘adults’ are used by authors in the description of the samples;
- Published in journals in or after 2000; and
- Written in English.

#### Exclusion criteria

- Informal literature review (review does not have defined research questions and does not have defined search process) or discussion papers;
- Studies with non-cancer diseases or mixed populations without provision of separate results for cancer patients; and
- Protocols, editorial comments, conference abstracts, guidelines, and policies

### Study selection and data extraction

The retrieved studies were exported into Endnote version X9, which was subsequently used to remove duplicates. Titles and abstracts of the remaining studies were screened by the first author (HAO) for eligibility against the inclusion and exclusion criteria. Full text of potentially eligible studies was then located for further screening. The second author (AM) was responsible for making the final decision of any uncertainly that the first author encountered during the assessment of full text papers. Reasons for excluding reviews were identified and documented.

For each included study, data were extracted by one author (HAB) and reviewed by a second author (AM) if needed. A data extraction sheet was utilised to record the following data; (1) citation details: authors, year of publication, and country of the first author; (2) number of studies included and sample descriptions; (3) aim(s) of the review; (4) results (main findings) summarised based on the SEM; (4.1) category of challenges; personal, system, policy and organisational factors, (4.2) category of possible facilitators; personal, system, policy and organisational factors (Table 2).

**Table 2:** Summary of aims and key findings of the included reviews

Author (Year), Implementation Year(s), Country/Region	Review aim	Setting/ Population	Findings	
			Barriers	Possible facilitators/recommendations for improvement
Donkor, Luckett, Aranda and Phillips [39], 1990–2017 Australia Systematic review, included 18 studies	To identify the facilitators and barriers to the implementation of cancer treatments and PC.	LMICs Cancer	Health system: • Drug importation process Policy: • Lack of financial support • Limited political commitment • Restrictive pharmacovigilance laws and regulations • Fragmented health system Organisation/ structure: • Limited physical infrastructure	Personal: • Education • Community sharing Health system: • Creating a learning environment • Information management system Policy: • Payment support • Stakeholder sharing • Political commitment • Positive relationships with international organisations • Strategy aligned with national policy
Soto-Perez-de-Celis [46] 2017 USA	To identify the existing deficiencies and providing a framework for the improvement of PC.	Latin America Cancer	Personal: • Cultural barriers Health System: • Lack of opportunities for clinical training Policy: • Inadequate or inappropriate legislation • Lack of comprehensive national PC plans • Unreliable reporting of data Organisation/ Structure: • Insufficient infrastructure	Personal: • Improve education • Enhance cultural aspects • Individualized care for patient’s preferences and beliefs Health System: • Increase the availability of pain medication • Training to all HCPs • Enhance, expand access to medication Policy: • Design comprehensive PC plans • Integrate end-of-life care into national health care laws • Enhance research Organisation/ Structure: • Improve infrastructure
Fadhil et al. [40] 2017 Egypt	To identify barriers to the development of PC.	Eastern Mediterranean Region Cancer	Personal: • Poor awareness of policy makers about PC • Poor awareness of HCPs about PC • Poor public awareness Health System: • Little partnership working • Insufficient PC education for HCPs • Gaps in access to essential pain-relief medicines. Policy: • Scarcity of national plans and policies • Complicated political situations • Weak health-care systems • Absence of PC in national policies	–
Ali [47] 2016 Kenya	To assess the integration of PC services into the public healthcare system	Kenya cancer	–	Health system: • Training HCPs • A higher diploma in PC Policy: • The government budget for PC services • Include PC in local health strategies and plans. • National PC guidelines
Hannon et al. [33] 2015 Canada	To overcome barriers that continue to affect the availability of PC in LMICs.	LMICs cancer	Personal: • Negative attitudes about PC and death and dying Health System: • Limited access to opioid medication • Lack of training of HCPs and volunteers Policy: • Lack of investment in health systems	Personal: • Education of HCPs • Shifts in societal norms to PC • Shifts in HCPs norms to PC Health System: • Changes in legislation restricting access to opioid medications • Training of health professionals; Policy: • A health policy that supports the integration of PC • Investment in systems of health care delivery • Development of rigorous data and research • International partnerships
Rochmawati et al. [48] 1990–2015 Indonesia Systematic review, includes 9 studies	To identify facilitators and barriers to the provision of PC.	Indonesia Cancer, HIV/AIDS	Personal: • Knowledge deficit and misunderstanding of HCPs Health System: • Difficult access to narcotic drugs Organisation/ Structure: • Geography	Personal: • Family and community support Policy: • Policy and organisation support Health System: • Volunteering
Abdel-Razeq et al. [32] 2014 Jordan	To discuss challenges and offer suggestions for the improvement of cancer management.	Jordan Cancer	Personal: • Negative HCPs attitudes • Negative public attitudes Health System: • Lack of specialized human resources • Lack of adequate training of responsible staff • Interrupted opioids supply and availability • Shortage of trained female nurses • Few specialized ancillary support personnel Policy: • Not available outcome data at a national level	Personal: • Increase HCPs knowledge Health System: • Structured training programs for HCPs Policy: • Integration of both clinical care and clinical research
Zeinah et al. [34] 2012 Qatar	To outline current PC at Middle Eastern countries. To address major challenges hindering the development of PC.	Middle East countries Cancer	Personal: • Lack of education and awareness Health System: • Shortage of specialized PC teams Policy: • Political issues • Scarcity of resources • Shortage or lack of funding • Lack or deficiency governmental support Organisation/ Structure: • No application of service (including opioid use and expertise)	Personal: • Raising awareness of the public on opiophobia; • Raising awareness of the HCPs on opiophobia. Health System: • Informal training to medical oncologists in PC. • Providing formal education to HCPs Policy: • Adequate funding for training programs.
Basu et al. [35] 2013 USA	To provide an overview of the progress in providing PC in low- and medium-resource countries. To present the development of PC in Ethiopia.	LMICs Cancer	Personal: • Negative cultural attitudes and beliefs of patients • Negative cultural attitudes of physicians Health System: • Lack of a trained workforce; • Lack of availability of opioids or restricting in their use Policy: • Lack of funding	–
Silbermann et al. [49] 2012 Israel	To address the accomplishments and challenges of palliative cancer care in Middle Eastern countries.	Middle East countries Cancer	Personal: • Families’ feeling of alienation and isolation • Families’ fear of neglect by the primary physician Health System: • Lack of relevant training of HCPs • Poor accessibility to essential PC drugs • Delay in referrals Policy: • Lack of health policies and plans	Personal: • Education of physicians and nurses about PC principles • A community-based orientation Health System: • Introduce PC principles into the curricula • Develop postgraduate training programs for physicians and nurses. Policy: • Public policy
Elcigil [50] 2011 Turkey	To assess the status of PC in Turkey.	Turkey Cancer	Personal: • Lack of PC education • Lack of public awareness • Limited knowledge of opioid analgesics Health System: • Lack of training programs • Shortage of nursing staff • Lack of certification for PC Nursing Policy: • Very limited research	Personal: • Increase public awareness channels Health System: • Disseminate information on certification of PC nurses to agencies. Policy: • Establish interdisciplinary research on PC concepts • Increase funding for research • Evidence-based curriculum to strengthen the teaching of PC concepts
Shawawra and Khleif [51] 2011 Palestine	To conduct a needs assessment survey within facilities that provide care for oncology patients in the West Bank.	Palestine Cancer	Personal: • Lack of community awareness on PC Health System: • No presence of educational resources for PC, • No training programs in PC, Policy: • An absence of organisational strategic planning, • No standards for PC service • An absence of national standards on PC.	Personal: • The need for public awareness. Health System: • The need for training of HCPs • Introduce PC principles into the curricula Policy: • Networking between the national non-governmental organization's and the Ministry of Health . • National policy and standards on PC and opioids legislations. • Baseline data and research. • Interdisciplinary teamwork.
Bingley and Clark [52] 2008 UK	To review PC development in six Middle East countries	Middle East countries Cancer	Personal: • Opioid phobia in the public • Opioid phobia in professionals • Lack of public awareness of PC • Lack of professional level awareness Health System: • Inadequate professional training programs Policy: • Lack of funds • Lack of government support.	Personal: • Public education programs; • Raising awareness about the need for PC Health System: • Increasing national and international training • Improving opioid legislation Policy: • Improving health care policies; • Negotiating for a secure government or health insurance funding provision
McDermott [53] 2007 UK	To identify strengths and weaknesses in the state of development across the subcontinent.	India Cancer	Personal: • Limited knowledge of patients about PC Health System: • Unavailability of opioid • Shortage of workforce Policy: • Limited national PC policy • Insufficient funding for services • Absence of social security system Organisation/ Structure: • Poverty; • Population density • Geography	Personal: • Increase public awareness of PC Health System: • Improve drug availability and expertise Policy: • Include PC in medical and nursing curricula • Design and implement a national PC policy

*LMICs* Low- and Middle-Income Countries; *UK* United Kingdom; *USA* United State America; *PC* Palliative Care; *HCPs* Health care Providers

### Quality assessment of the reviews

Two authors independently used the Assessment of Multiple Systematic Reviews (AMSTAR-2) tool for systematic review articles or the International Narrative Systematic Assessment (INSA) tool for narrative review articles [54] to assess the quality of all included review articles. AMSTAR-2 has 16 items; each item is rated as ‘yes’ for a positive result or ‘no’ for no information provided or ‘partial yes’ response in some cases where we consider it useful to determine partial compliance with the standard. The overall quality of a systematic review has been rated as ‘high’, ‘moderate’, ‘low’ and ‘critically low’ [55]. High quality means that the paper provides comprehensive summary of the results of the available studies; moderate-quality indicates that the review paper has more than one weakness, but no critical flaws; while low quality shows that the paper has a critical flaw and may not provide an accurate and comprehensive summary of the available studies [55]. INSA contains seven items which include clarity of background, objective, conclusion, description of selection of studies, study characteristics, results, and conflict of interest. Each item is graded as ‘yes’ or ‘no’ and one point is given for each of the seven criteria. A review with a total INSA score of ≥5 points is considered a ‘good’ quality review [54].

### Data analysis

As mentioned, the SEM was selected as an analytical framework for data analysis (Fig. 1). The McLeroy et al. model that is generated from the SEM was adopted to align the context and to conceptualize the review findings [42]. This is done to remove distinction between intrapersonal and interpersonal domains and, in its place, differentiate between HCP, patient and caregiver domains. Furthermore, the model is appraised to identify community domains as health system domains [42]. Therefore, the final modified model has four levels; 1) personal level; 2) organisational level; 3) health system level; 4) policy/payment level. The personal level focuses on patients, families, and HCPs, while the organisational level deals with the culture of the organisation and infrastructure. The health care system level describes workforce and training, and finally the policy/payment level relates to legislation and funding.

## Results

### Characteristics of the included reviews

**Fig. 2:**
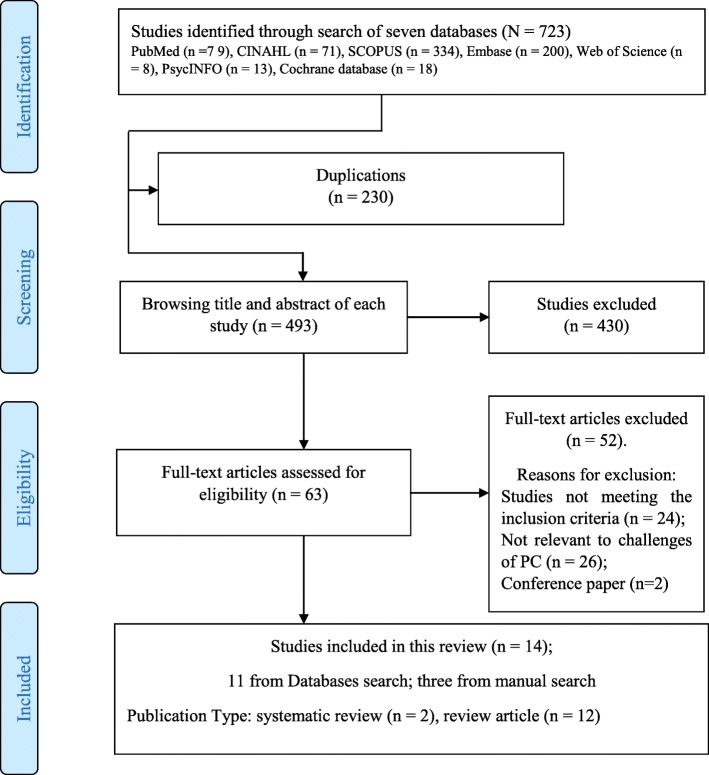
Flow Diagram of the identification of papers

The review yielded 723 articles, and 11 met inclusion criteria. Each review reference list was further assessed to see if any relevant review has been omitted. Through this, an additional three reviews were added. Consequently, 14 reviews are included for analysis (Fig. 2).

The majority of the included studies (*n* = 12) were narrative reviews [32–35, 40, 46, 47, 49–53], and the remaining two were systematic reviews [39, 48]. Concerning the geographical focus of the reviews, reviews (*n* = 4) originated from Middle Eastern countries [34, 40, 49, 52] and LMICs (*n* = 3) [33, 35, 39]. The other seven reviews focused on specific countries including Jordan [32], Latin America [46], Indonesia [48], Turkey [50], India [53], Palestine [51], and Kenya [47].

With regards to the origin of the authors, seven authors were from developed countries [33, 35, 39, 46, 49, 52, 53]; including two from the USA [35, 46], two from the UK [52, 53], and one each from Australia [39], Canada [33] and Israel [49]. As for the year of publication, the number of publications increased significantly in the last 4 years [32, 33, 39, 40, 46–48]. Only two reviews were published before 2010 [52, 53].

In terms of disease category, 13 reviews focused on patients diagnosed with cancer [32–35, 39, 40, 46, 47, 49–53], and one review focused on mixed chronic diseases, including cancer [48]. Concerning the number of studies included in each review, only two reviews included the number of studies in their reviews [39, 48]; one of them included nine studies [48], and the other [39] included 18 studies. Table 2 displays the characteristics and main findings of all the included reviews.

### Quality of the included studies

Generally, the methodological quality of the narrative reviews was good. Nine narrative reviews scored ≥5 points on the INSA tool, reflecting good quality reviews [32–35, 40, 46, 49, 51, 52]. The rest of the reviews (*n* = 3) had a score equal to 4 points on the INSA tool [47, 50, 53]. Half of the narrative reviews did not report a conflict of interest [35, 47, 50–53]. The methodological quality of the systematic reviews [39, 48] was high, which suggests a paper presenting an accurate and comprehensive summary of the results of the available studies that address the question of interest.

### Challenges to the provision of palliative care

#### Personal challenges

Personal challenges focused on knowledge, attitudes, beliefs, skills, culture of patients and families, the general public and HCPs. Seven reviews [34, 40, 48, 50–53] showed that knowledge deficits of HCPs about PC and the use of opioid analgesics were the most common challenges affecting the provision of PC for cancer patients. Additionally, some patients could not distinguish between PC and hospice care [34, 48, 50, 52]. Five other reviews indicated that HCPs, families, and the general public were poorly aware about PC and its benefits to patients and health-care systems [34, 40, 50–52]. Besides, four reviews pointed to negative attitudes and beliefs among HCPs as obstacles in the provision of PC services [32, 33, 35, 49].

#### Health care system challenges

Health care system challenges included workforce development issues, education, service delivery, and access issues across organisations. Thirteen reviews discussed the health care system as a barrier to the provision of PC [32–35, 39, 40, 46, 48–53]. Shortage of or inadequately trained PC workforce was the most critical barrier to the provision of PC [32–35, 50]. This includes a shortage of nursing staff [50], especially the shortage of trained female nurses [32], and few specialised ancillary personnel [32]. Moreover, there was a lack of professional training programmes for HCPs [46, 49–51], including the failure to offer basic training to staff [32] and little collaboration/partnership between health organisations [40].

Drug restrictions were also identified as one of the health care system challenges, as reported in seven reviews. These restrictions included inadequate access to essential pain-relief medicines [33, 35, 40, 48, 49], interruption of opioids supply and availability [32, 35], and cumbersome drug importation processes [39].

#### Organisational challenges

Organisational level issues shed light on structure, organisational culture, policies, and procedures of the organisation. Four reviews reported organisational-related issues as a barrier to the provision of PC in LMICs [34, 39, 46, 53]. Facilities infrastructure constitute one of the major components of the health care system and this must be estimated and planned before the provision of any services. Limited physical infrastructure (i.e., buildings, equipment and supplies, beds, chairs, etc) were reported as the critical challenges to the provision of PC [39, 46]. In addition, the geography of the country [48, 53] (i.e., people living in a rural or remote area) could hinder access to PC services [53].

#### Policy/payment challenges

Twelve reviews reported key policy challenges (funds, legislation, and research) as factors impeding the provision of PC for cancer patients [32–35, 39, 40, 46, 49–53]. Across five reviews, shortage or lack of funding was recognised as the most critical barrier to the provision of PC [34, 35, 39, 52, 53]. Several other studies acknowledged that lack of a comprehensive national PC plan [40, 46, 49, 51], inadequate or inappropriate legislation and policy [46, 51, 53], fragmented or weak health care system [39, 40], and lack of government support [34, 52] negatively impacted on the delivery of PC to cancer patients. Collectively, these identified challenges were influenced by limited political commitment [39], complicated political situations [40], restrictive pharmacovigilance laws and regulations [39], or absence of a state-sponsored social security system [53].

#### Possible facilitators for the provision of palliative care

Of the 14 reviews, 13 mentioned facilitators for overcoming challenges associated with the provision of PC. Personal facilitators were discussed in 11 review articles [32–34, 39, 46, 48–53]. Health care system facilitators were also presented in 11 reviews [32–34, 46–53]. Policy facilitators were enumerated in 12 reviews [32–34, 39, 46–53], while organisational facilitators were discussed in only one review [46].

### Personal facilitators

Reviews indicated that adequate and continuous education is needed for both HCPs and patients and the wider general public [32–34, 39, 46, 48–53] for changing their attitude to PC and improving their awareness of PC [34, 48, 50–52]. Adequate education of the general public and family were covered in 10 reviews [32–34, 39, 46, 48–50, 52, 53], appropriate education of HCPs in four reviews [32–34, 51], improving public and HCPs attitudes on opiophobia in two reviews [34, 49], and enhancement of cultural aspects and providing PC that valued patient’s preferences and beliefs were described in one review [46].

### Health care system facilitators

Facilitators related to the health care system were mentioned in 12 reviews [32–34, 39, 46–53]. Adequate training of HCPs was also identified as a critical health care system facilitator that could not only improve the quality of care but also increase the workforce [32–34, 46, 47, 51, 52]. Moreover, the quality of the workforce can be enhanced through increasing national and international professional programmes [47, 49, 51, 52], providing informal training to medical oncologists [34], creating a supportive learning environment for HCPs and developing information management systems [39], and integrating PC into curricula and practice [47, 49–51, 53]. Other facilitators identified included changing legislation that inappropriately restricts access to opioid medications [33, 46, 52, 53] and improving access to and availability of narcotic drugs [46, 53].

### Organisational facilitators

Improving the physical infrastructure of health care settings can play a crucial facilitative role in the development and provision of PC, as reported in two reviews [46, 51].

### Policy/payment facilitators

Policy/payment issues were the main facilitators to the provision of PC for cancer patients, as described in 12 reviews [32–34, 39, 46–53]. Designing and implementing a national PC policy were the main facilitators discussed in nine reviews [33, 39, 46–49, 51–53]. These can be achieved through the involvement of stakeholders [39], budget support [33, 34, 39, 47, 50], and negotiating for secure government or health insurance funding provision [52]. Enhancing and increasing research about PC were also identified as essential policy facilitators [32, 46, 50, 51], which help in identifying the needs and gaps in the provision of PC.

## Discussion

This systematic review adds to the literature on the topic by providing a systematic and more comprehensive mapping of the challenges associated with the provision of PC services in LMICs. This goes further to identify some common facilitators to overcome these challenges in LMICs. Fourteen reviews have highlighted that the provision of PC for cancer patients in LMICs are affected by a wide range of challenges. Personal and health care system-related issues have been highlighted as key challenges to the provision of PC. One interesting finding was that although the included reviews focused on LMICs, seven authors who conducted these reviews were from developed countries, indicating the interest from developed countries about PC in countries with minimal resources and in developing economies.

Overall, insufficient knowledge, poor awareness, negative attitudes and beliefs of patients, families, the general public, and HCPs are crucial personal challenges to the provision of PC in LMICS. Four reviews that were conducted in developed countries [41, 56–58] reported similar findings. Furthermore, a population-based study suggests that a low level of awareness and knowledge deficit common among adults are part of the challenges [59]. Another study linked insufficient knowledge and low level of awareness of patients, families, the public, and HCPs to PC services and their benefits [60] to these challenges, potentially contributing to delayed referrals of patients to PC services [61–63]. Therefore, PC education has been recommended as the first step [64] to increase awareness, promote positive attitudes and improve knowledge about these services among the general public and HCPs [65–67]. This can be realised through integration of core competencies of PC into the curricula of universities [64, 68] and cultivating more positive attitudes in the general public through the media or public engagement programmes.

The results of this review indicate that shortages of or inadequately trained workforce, and poor accessibility and availability of pain-relief medication are essential health care system challenges to the provision of PC. There is a significant shortage of specialised HCPs in the PC world [69, 70]. This shortage will affect the quality of the PC services provided [71] and the fulfilment of expectations of cancer patients [71]. Investment in terms of time and resources in the training of competent a PC workforce is a recommended facilitator in addressing the workforce shortages. Also, volunteers can play crucial roles in supporting the health of cancer patients and overcoming workforce shortages [72, 73]. There are benefits in involving and utilising volunteers in health settings. They can be used for caring and delivering support and services to patients and the overall economy of the health care system [74, 75]. Using volunteers to improve psychosocial health, education, and engagement might be an effective way for lowering costs and the economic burden of delivering PC services in LMICs [76]. Volunteers, however, require effective and appropriate training to enhance their performance and the quality of care provided to patients [38, 77]. This has been successfully implemented in some LMICs [78].

Poor accessibility of pain-relieving medications is a unique barrier to accessing PC in LMICs, with 80% of people having little or no access to such medication [38]. Despite the availability of pain-relieving medications as a basic component of health care systems, false perceptions of patients and their families [79–82] and HCPs [83, 84] can be major challenges to the provision of PC. Many patients avoid using pain-relieving medication because of their belief and fear that the use of this medication will lead to addiction [79–82]. While HCPs may not prefer to prescribe these medications because of their lack of adequate pain assessment skills and their beliefs also that the use of opioids can cause addiction [83, 84]. Besides the perceptions of patients and HCPs about the use of opioids, it is further acknowledged that developing countries constitute 80% of the world population but receive only 6% of the available morphine [85]. The European Society of Medical Oncology, through its Global Opioid Policy Initiative project, has identified a range of issues impacting the use of opioids in LMICs, and these include not only unavailability of opioids, but also outdated policies that discourage access, limited awareness and unnecessary administrative obstacles, and inadequate education and empowerment of HCPs [86]. Often it is a combination of all above factors that impede the use and uptake of opioids in LMICs making access to such medication a complex issue. Several authors recognise the worldwide lack of access to opioids [87–89] as a factor affecting the provision of PC to cancer patients. Furthermore, for PC to become readily available, restrictions on the access to opioid drugs in LMICs should be removed [90]. This is because opioid therapy requires both availability and affordability for cancer patients while receiving PC, as pain management is one of the critical components in PC services [64, 84]. A few authors opine that the availability and affordability of opioids are essentially part of “human rights” [91, 92], and WHO has developed a list of essential medicines for a basic healthcare system, including opioids and medicines for other common symptoms in PC [93]. Legal restrictions, such as national laws often restrict opioid use or prohibit access to narcotics [87, 89, 94, 95]. These impediments, nonetheless, have negative consequences on patients and their families [96]. Reviewing or changing related legislation and policies are needed to overcome these impediments. This can be carried out at the national level by analysing legislation and policy documents [87, 95, 97].

Limited physical infrastructure, in addition to the geography of the country, poverty, and population density are the main organisational challenges to the provision of PC. Developing countries experience financial/funding challenges and poverty, which negatively affect the development of their health care systems [98]. Therefore, it is recommended that policy makers collaborate with national and international organisations to secure funding for improving health care provision.

Most people in need of PC are at home due to transportation difficulties or limited income to with accessing care or buying medication [99]. Integrating PC into primary care services is a recommended strategy to improve access to PC for patients living in remote areas [100]. This integration will help patients and their families, who are living in remote areas, to receive comprehensive care without being overwhelmed by personal cost issues [101].

Shortage of funding and lack of a comprehensive national plan on implementing PC that are identified in this review are complex and overlapping. Lack of national plans and policies on the provision of PC services are common in most developing countries [28]. LMICs should develop national PC plans and integrate these plans within their strategy for non-communicable diseases. This should be in line with the local context and health care needs and can be achieved through engagement of policy makers and budget estimations [102]. There was also lack of cost data available in the included reviews and previous literature which is also considered a barrier to introducing and estimating PC cost. In the reviews assessed, most LMICs only briefly highlighted the insufficient funding and limited-resources, although availability of funding is of paramount importance in the development of PC services.

For overcoming policy challenges, policy makers should understand the processes involved in PC implementation, factors that affect implementation and the introduction of solutions for overcoming these obstacles [103]. Credible setting of policy agendas, realistic policy formulation, timely policy implementation, and policy monitoring and evaluation [104] remain pressing needs of LMICs for overcoming these impediments. Involving policy makers in this process is essential and has a significant positive effect on defining their priorities, diagnosing their challenges, and implementing appropriate solutions for service improvement [105].

Challenges that hinder the provision of PC differ significantly in developed and developing countries in terms of scope, context, culture, and religious beliefs. However, there are some similarities in challenges to PC provision. The main common obstacles to the provision of PC in both developed countries and LMICs include lack of a properly trained workforce [41, 56–58], fear among HCPs [106, 107], lack of awareness about PC [59, 60, 108–110], limited funding and lack of coordination amongst services [106]. However, in developing countries, factors affecting the provision of PC services to cancer patients further include lack of resources and inadequate physical infrastructure. Others are related to administrative challenges, which are mainly centred on access to opioids and organisational commitment [31]. Furthermore, culture, beliefs, and norms about death and opioid consumption are working against PC in LMICs. Compared to developed countries, inadequate accessibility and availability of opioids are unique to developing countries.

About 83% of people in developing countries consume only 9% of the world morphine available [111], in comparison with 7.5% of the countries located in the American and European Regions that have adequate consumption levels of opioids [112]. A lower percentage of consumption of opioids in developing countries may be tied to the legislation in some countries whereby patients are required to register to receive opioids. In fact, some African and European countries even require special registration for hospice patients [87, 95].

Negative attitudes about PC and death, opioid phobia, and families’ feeling of alienation and isolation are the most common challenges in developing countries, while, misperceptions equating “PC” with end-of-life care of HCPs and the general public are the main challenges in developed countries [41, 56–58]. Furthermore, in developed countries, PC is provided relatively well for cancer patients, and most patients enjoy good access to services [31]. Nevertheless, uneven PC coverage [106] is most common in countries with low-resources.

Future research efforts are needed to develop a body of evidence that is adequate to support effective learning and policy development. Furthermore, other potential challenges that may hinder the provision of PC that have not been covered in this review may form the basis for future studies. For instance, two significant aspects may be considered. The first aspect is the HCP's voice. As most reviews have focused on knowledge, attitudes, and beliefs, none has investigated communication competencies between HCPs and their relationship with their patients, an important aspect for the successful provision of PC. Communicating professionally with patients improves their attitudes [113–115]. The second aspect is the patients’/family voice. Most reviews have examined the attitudes of patients and their families towards PC services, but none studied the priorities, needs, and wishes of patients about PC services in LMICs. For policy development, assessing the country readiness for the provision and integration of PC is an essential step to an effective adoption [116].

### Strengths and limitations

A strength of this systematic review is that it covered several databases, with up to date data to capture information more relevant to current health care systems. However, this review also presents with several limitations, such as limited number of original studies included in the reviews, focusing only on reviews, and the narrative format of most reviews used in the present analysis.

## Conclusion

This review expands the existing knowledge about challenges and possible facilitators on the provision of PC services for cancer patients in LMICs. Understanding these challenges from each level (from policy and organisation down to individual patient-health care providers) could help in the development of PC pathways in LMICs and it could be used as a guide to develop a model for the provision of PC services. It can be used by policy makers to understand the implementation of a new PC programme in their countries and the areas they need to focus on and prioritise. It can be used by non-governmental organisations to supplement governmental efforts and cover related gaps. Also, political influence and support are required to ensure sustainability and high-quality PC services. Although PC for patients with cancer is gaining gradual recognition worldwide [24], it still lacks widespread adoption in LMICs. Overall, this detailed analysis of challenges and possible facilitators’ offers the opportunity to develop interventions to improve and implement PC into health care systems in LMICs.

## Data Availability

Data used for analysis in this review are all extracted from the original published reviews and are presented in Table ​2 (Summary of the included reviews’ aims and key findings).

## Abbreviations

AMSTAR: Assessment of Multiple Systematic Reviews
CINAHL: Cumulative Index to Nursing and Allied Health Literature
MECs: Middle East Countries
HCPs: Health Care Providers
INSA: International Narrative Systematic Assessment
LMICs: Low- and Middle-Income Countries
MeSH: Medical Subject Heading
PC: Palliative Care
PRISMA: Preferred Reporting Items for Systematic Reviews and Meta-Analyses
QOL: Quality of Life
SEM: Socio-Ecological Model
UK: United Kingdom
USA: United State America
